# Adaptive Unscented Kalman Filter for Target Tacking with Time-Varying Noise Covariance Based on Multi-Sensor Information Fusion

**DOI:** 10.3390/s21175808

**Published:** 2021-08-29

**Authors:** Dapeng Wang, Hai Zhang, Baoshuang Ge

**Affiliations:** 1 School of Automation Science and Electrical Engineering, Beihang University, No. 37 Xueyuan Road, Haidian Distirct, Beijing 100083, China; wangdapeng@buaa.edu.cn; 2 Science and Technology on Aircraft Control Laboratory, Beihang University, No. 37 Xueyuan Road, Haidian Distirct, Beijing 100083, China; 3 Yancheng State-Owned Assets Investment Group Co., Ltd., No. 669 Century Avenue, Yandu District, Yancheng 224000, China; gebaoshuang@buaa.edu.cn

**Keywords:** multi-sensor information fusion, process-error estimation, adaptive and robust unscented Kalman filter, target tracking

## Abstract

In this paper, an innovative optimal information fusion methodology based on adaptive and robust unscented Kalman filter (UKF) for multi-sensor nonlinear stochastic systems is proposed. Based on the linear minimum variance criterion, this multi-sensor information fusion method has a two-layer architecture: at the first layer, a new adaptive UKF scheme for the time-varying noise covariance is developed and serves as a local filter to improve the adaptability together with the estimated measurement noise covariance by applying the redundant measurement noise covariance estimation, which is isolated from the state estimation; the second layer is the fusion structure to calculate the optimal matrix weights and gives the final optimal state estimations. Based on the hypothesis testing theory with the Mahalanobis distance, the new adaptive UKF scheme utilizes both the innovation and the residual sequences to adapt the process noise covariance timely. The results of the target tracking simulations indicate that the proposed method is effective under the condition of time-varying process-error and measurement noise covariance.

## 1. Introduction

There have been increasing demands for developing robust, adaptive, and accurate multi-sensor information filters (MSIF), which have been widely applied to many fields such as navigation systems, modern industries, military threat detection, target tracking, and remote sensing [1,2]. Especially in recent years, there have been many state estimation problems in which the processes were often non-linear and uncertain for tracking and navigation, for example [3]. Hence, the theory has been researched and broadly applied to many realistic systems. A method with both adaptability and robustness cannot be realized in real time for a single sensor/observer system [4]. By using a multi-sensor structure, an information fusion algorithm can obtain much more accurate estimations than a single one [5,6]. However, to the best of our knowledge, few methods focus on both adaptability and robustness in MSIF. From a system point of view, there are mainly two different methods to process the data from a multi-sensor [7]. The first method is the centralized filter where all raw sensor data are fed to a central site for processing [1]. The second one is the decentralized or distributed filter where the process is divided between some local filter concurrently to obtain individual raw data-based estimates and one master/center filter to fuse those local estimates to provide a much accurate global optimal estimate [6,8].

The centralized filter is also called measurement fusion, because all observations are directly fused to obtain a final estimate. The main advantage is high accuracy due to the use of lossless information. However, in practice, this architecture is heavily challenged by a large complexity of computation while the number of sensors increases. Another drawback of this method is the lack of robustness when one or more sensors fail.

In the decentralized structure, the complexity of computation can be reduced in the center filter because part of the computation is taking place at the local filters; furthermore, fault detection and isolation are easier to be implemented. For the reasons given above, parallel structure, which can provide improved reliability and fault tolerance of sensors, has been paid more attention to and implemented in many aspects [1,8].

Frank et al. proposed estimators for multi-sensors with different failure rates [9]. They took the stochastic perturbations and the probability of sensors’ failure into account, so the robustness could be improved. However, the matrices which signify the direction of perturbations are assumed known, which may not be available in practice for many systems. An excellent piece of literature also focused on the problem, where the Bernoulli random variables were used to predict the phenomenon of missing observations [10]. Unfortunately, the assumption that any failed sensor may recover after specific *m* instances of time cannot be held in many harsh environments.

Qiu et al. proposed a diagonal weighting matrix method for the fusion of local estimates [7]. However, this algorithm gains computational efficiency at the expense of a loss in accuracy [11]. Zhang et al. put forward a method to fuse the multi-sensor measurements in a sequential way [12]. In [13], under the energy harvesting constraints, a robust fusion filtering over a multi-sensor system is proposed. By using the covariance intersection fusion strategy, this theoretical framework for discrete time-varying stochastic multi-sensor systems is established. Based on the fact that so many scholars successfully use the decentralized structure to improve the performance of multi-sensor information fusion, this paper adopts the decentralized structure [8].

For a local filter in the MSIF, there are several options. Kalman filter is a promising method for linear problems [14]. When problems become nonlinear, a set of improved forms could be adopted, of which the extended Kalman filter (EKF) and the unscented Kalman filter (UKF) are used widely. Using Taylor series expansions, the EKF linearizes the non-linear models to make them convenient for the standard Kalman filter procedure. The core drawback of the EKF makes it unable to achieve sufficient estimation accuracy for a maneuvering target, because the first-order approximate error could be huge under strong nonlinear conditions. Although a lot of efforts were made to estimate the states by the adaptive algorithms based on the covariance estimation, as argued by Ge et al. [15], the EKF and its adaptive forms are still not optimal options for the key reason above. The generic particle filter (PF) and the cubature Kalman filter (CKF) are also well-known methods for nonlinear problems, as mentioned by [16]; the high computation cost might not be affordable in many applications.

The posterior mean and covariance of any Gaussian random variable in third-order accuracy could be approximated by UKF based on the unscented transformation (UT) [17]. To apply the UKF, process-error and measurement noise should be taken into consideration, so plenty of adaptive UKF methods have been proposed. Soken et al. corrected the mismatches of the process noise covariance (Q-adaptation) based on an adaptive UKF algorithm, and they applied the method to accomplish the picosatellite attitude estimation under the condition that the process noise covariance may vary [18]. However, by using one scalar parameter to correct the Q, the accuracy is limited to a certain extent. Similar to [18], Chang proposed a method with both adaptivity and robustness [4]. The method can deal with the condition that both the process and measurement noise covariance change at the same time, but not in real time. Meanwhile, the drawback of the heuristic method is the same as [4], that only one scalar was calculated for adapting the Q or R, which stand for the measurement noise covariance.

As argued in [10], if measurements only contain noise, they can be seen as outliers [6]. To make a more accurate estimation of the noise covariance, plenty of scholars have turned to adaptive UKF methods. Mohamed et al. developed an adaptive Kalman filter, based on the maximum likelihood criterion for the proper choice of filtering weight. They argued that the method is efficient by adapting the matrices R and/or Q [19]. The basic idea of [19] is to adapt the R by innovation sequences and the Q by residual ones. However, the innovation and residual sequences obtained from the filter are not independent [15].

In [20], based on an adaptively robust EKF, Yuan et al. proposed a PDR/UWB (Pedestrian Dead Reckoning and Ultra-Wide Band) integrated navigation algorithm. To obtain the adaptability, the algorithm takes the positioning scene and the heading as constraints. The robustness of the algorithm is achieved by adopting the idea similar to [6]. However, in many other applications such as tracking and remote sensing, the constraints are not always available or difficult to implement because of the great complexity and the high computational cost [21]. In [22], to handle measurement outliers, the robust estimation reduces the weight of the observation; to handle the model error, an adaptive factor is introduced to balance the adverse effect. This algorithm has inspired many scholars. In [23], two novel quantitative nonlinear observability measures are proposed to get an optimal filter design. However, both [22] and [23] inevitably use the state-dependent calculations to adjust the measured values or the weight matrix, so the same problem as in [20] mentioned above exists. In [24], an adaptive filtering method was proposed. The authors used the residual error to construct a low-pass filter together with the process noise covariance. They argued that the high process noise could be effectively suppressed. However, if measurement noise covariance is not estimated accurately in a timely manner, the residual error will be inaccurate; then, in the next iteration, the residual error may not be adjusted only by the algorithm. So, it is necessary to estimate the measurement noise covariance as accurately as possible. Moreover, it is better that the estimation of the measurement noise covariance is independent of the process noise or estimation of the state [15].

If the estimation of the noise covariance matrix R could be accurately made, it would be a solid foundation to give the matrix Q a relatively accurate estimation. Zhang et al. developed a measurement-based adaptive Kalman filtering algorithm (MAKF) that overcame the instability drawback of improved Sage–Husa adaptive filter for the integrated navigation system [25,26]. Realizing the limitation of MAKF is that the following assumption could not always be held—one of the measurement noise covariances is relatively smaller than the other—the group of Zhang afterwards developed an improved method named redundant measurement noise covariance estimation (RMNCE) [27], which can estimate the noise variance of the measurement and is not affected by the process state estimation error. So, in this work, we utilize the RMNCE, which can deal with the unknown noise covariance in real time, to calculate the measurement noise covariance ${\hat{R}}_{k}$ for each sensor.

In our proposed method, the matrix ${\hat{R}}_{k}$ of each sensor is also calculated through innovation sequences, denoted as $R_{in}$, as mentioned in [28]. We denote the ratio between $R_{in}$ and ${\hat{R}}_{k}$ as the indicator to reflect whether there would be non-ignorable process error or not. Furthermore, if the indicator or the hypothesis test theory based on chi-square suggests the existence of process error, the trigger for adaptation is on. To the best of our knowledge, the combination of the two criteria is firstly introduced by this paper.

For a given MSIF problem, without loss of generality, the statistical properties of measurement noise are not reliable, though they could be obtained in advance. So, we adopt the RMNCE method to estimate the variance of measurement noise in multi-sensor system. Additionally, taking all the above adaptive Q estimation algorithms into consideration, a new Q estimation algorithm based on both innovation and residual sequences is given, which is inspired by [16]. Finally, the decentralized architecture is used to fuse the estimations from local sensors.

The contributions of the paper are twofold.

Firstly, an efficient algorithm is proposed for the MSIF to detect the process-error based on the indicator, which is combined by hypothesis test theory with the Mahalanobis distance of innovation sequences and the RMNCE. Then, an innovative Q estimation algorithm is proposed by using both innovation and residual sequences.

Secondly, to the best of our knowledge, the RMNCE algorithm together with a weighted factor is first introduced into MSIF. To begin with, the covariance of measurement noise obtained by RMNCE is not only used as the measurement noise covariance estimation of each sensor but also as the element to calculate the weighted factor. Then, a novel method is proposed to simplify the calculation of the weighted factor as an alternative to optimal matrix weights in [1]. Moreover, an indicator is also proposed based on the RMNCE to detect whether the process-error exists or not. At last, the simulation results demonstrate that the proposed scheme can increase the tracking precision with both adaptivity and robustness.

The remainder of this paper is organized as follows: in the next section, we describe the standard UKF, the RMNCE, an innovative adaptive UKF (AUKF) proposed by this paper and the decentralized MSIF. Section 3 introduces the adaptive multi-sensor information fusion algorithm (RAUKF-MSIF). Section 4 provides the simulation and discussions. Section 5 finally draws the conclusions.

## 2. The Decentralized MSIF and the Proposed Adaptive UKF

Considering a discrete time nonlinear stochastic system with *l* sensors, the process and measurement models can be described as

(1) $$\left\{ \begin{array}{l} X_{k}=f(X_{k-1})+\Gamma_{k-1}W_{k-1}\\ Z_{k}^{i}=h^{i}(X_{k})+V_{k}^{i},\;i=1,\;2,\;\ldots ,\;l \end{array}\right.$$

where $X_{k}\in {\mathbb{R}}^{n\times 1}$ denotes the state vector, $Z_{k}^{i}\in {\mathbb{R}}^{m_{i}\times 1}$ is the measurement collected by sensor i at sampling time instant k, $W_{k-1}$ and $V_{k}^{i}$ are uncorrelated zero-mean Gaussian white noise with compatible dimension [4,16,25,26,28,29], f(•) and h(•) are the known time-varying nonlinear state transition and measurement function, respectively. $\Gamma_{k-1}$ is the system noise-driven matrix with compatible dimension.

The statistical properties assumed about noise processes can be summarized as [4]

(2) $$\{\begin{array}{c} E\left[W_{k}W_{j}^{T}\right]=Q_{k}\delta_{k,j}\\ E\left[V_{k}^{i}V_{j}^{i}{}^{T}\right]=R_{k}^{i}\delta_{k,j}\\ E\left[W_{k}V_{j}^{i}{}^{T}\right]=0 \end{array}$$

where $\delta_{k,j}$ denotes the Kronecker delta function.

The following assumptions are also made as initial value

(3) $$E\left(X_{0}\right)=\mu_{0},\;E\left[\left(X_{0}-\mu_{0}\right)\left(X_{0}-\mu_{0}\right)\right]=P_{0}$$

where the initial state $X_{0}$ is independent of $W_{k-1}$ and $V_{k}^{i}$, $P_{0}$ is the initial estimation error covariance matrix.

In the following Section 2.1, the procedure of the standard UKF is briefly reviewed. In Section 2.2, the RMNCE is introduced. To improve the adaptivity of the estimation, the process-error should be detected timely, so an innovative adaptive UKF is proposed in Section 2.3. In Section 2.4, the structure of MSIF adopted by our work is given.

### 2.1. The Standard UKF

The UKF uses the fact that it should be easier to estimate a nonlinear distribution than to give an approximation of a nonlinear system [29]. In the standard UKF, to generate the sigma points to undergo the nonlinear transformation and calculate the first two moments of the transformed set, the UT, a deterministic sampling technique, is implemented. For the sake of simplicity, only one sensor of (1) is taken into consideration, and the general procedures are as follows:

Step 1: Initialization.

(4) $$\left\{ \begin{array}{l} {\hat{X}}_{0}=E\left[X_{0}\right]\\ P_{0}=E\left[\left(X_{0}-{\hat{X}}_{0}\right){\left(X_{0}-{\hat{X}}_{0}\right)}^{T}\right] \end{array}\right.$$

where ${\hat{X}}_{0}$ is the initial state, $P_{0}$ is the initial estimation error covariance.

Step 2: Sigma points generation.

(5) $$\left\{ \begin{array}{l} \chi_{k-1}^{\left(0\right)}={\hat{X}}_{k-1}\\ \chi_{k-1}^{\left(i\right)}={\hat{X}}_{k-1}+\sqrt{(n+\lambda )P_{k-1}},\;i=1,\;2,\;\ldots \;,\;n\\ \chi_{k-1}^{\left(i\right)}={\hat{X}}_{k-1}-\sqrt{(n+\lambda )P_{k-1}},\;i=n+1,\;n+2,\;\ldots \;,\;2n \end{array}\right.$$

where n denotes the dimension of the state; $\lambda =\alpha^{2}\left(n+\kappa \right)-n$ is the composite scaling parameter that is used for fine tuning, α is set to 0≤α≤1 and a good default setting on κ is κ=0 [15].

Step 3: State prediction.

(6) $$\left\{ \begin{array}{l} \chi_{k/k-1}^{\left(i\right)}=f\left(\chi_{k-1}^{\left(i\right)},k-1\right),\;i=0,\;1,\;2,\;\ldots \;,\;2n\\ {\hat{X}}_{k/k-1}=\sum_{i=0}^{2n}\omega_{i}^{\left(m\right)}\chi_{k/k-1}^{\left(i\right)}\\ P_{XX}=\sum_{i=0}^{2n}\omega_{i}^{\left(c\right)}\left(\chi_{k/k-1}^{\left(i\right)}-{\hat{X}}_{k/k-1}\right){\left(\chi_{k/k-1}^{\left(i\right)}-{\hat{X}}_{k/k-1}\right)}^{T}\\ P_{k/k-1}=P_{XX}+\Gamma_{k-1}Q_{k-1}\Gamma_{k-1}^{T} \end{array}\right.$$

where ${\hat{X}}_{k/k-1}$ is the predicted state mean; and $P_{XX}$ is the predicted state covariance. $\omega_{i}^{\left(m\right)}$ and $\omega_{i}^{\left(c\right)}$ are weights, which are defined as

(7) $$\begin{array}{l} \omega_{0}^{\left(m\right)}=\frac{\lambda }{n+\lambda }\\ \omega_{0}^{\left(c\right)}=\frac{\lambda }{n+\lambda }+\left(1-\alpha^{2}+\beta \right)\\ \omega_{i}^{\left(m\right)}=\omega_{i}^{\left(c\right)}=\frac{\lambda }{2\left(n+\lambda \right)},\;i=1,\;2,\;\ldots ,\;2n \end{array}$$

where β≥0 is used to incorporate the higher order information of the distribution, according to [15], for Gaussian distribution β=2 [29].

Step 4: Observation prediction.

(8) $$\begin{array}{l} \gamma_{k/k-1}^{\left(i\right)}=h(\chi_{k/k-1}^{\left(i\right)},k),\;i=0,\;1,\;2,\;\ldots ,\;2n\\ {\hat{Z}}_{k/k-1}=\sum_{i=0}^{2n}\omega_{i}^{\left(m\right)}\gamma_{k/k-1}^{\left(i\right)}. \end{array}$$

Step 5: Kalman gain calculation.

(9) $$\begin{array}{l} P_{XZ}=\sum_{i=0}^{2n}\omega_{i}^{\left(c\right)}\left(\chi_{k/k-1}^{\left(i\right)}-{\hat{X}}_{k/k-1}\right){\left(\gamma_{k/k-1}^{\left(i\right)}-{\hat{Z}}_{k/k-1}\right)}^{T}\\ P_{ZZ}=\sum_{i=0}^{2n}\omega_{i}^{\left(c\right)}\left(\gamma_{k/k-1}^{\left(i\right)}-{\hat{Z}}_{k/k-1}\right){\left(\gamma_{k/k-1}^{\left(i\right)}-{\hat{Z}}_{k/k-1}\right)}^{T}+R_{k}\\ K_{k}=P_{XZ}P_{ZZ}^{-1}. \end{array}$$

Step 6: State estimation and error covariance matrix update.

(10) $$\begin{array}{l} {\hat{X}}_{k}={\hat{X}}_{k/k-1}+K_{k}\left(Z_{k}-{\hat{Z}}_{k/k-1}\right)\\ P_{k}=P_{k/k-1}-K_{k}P_{ZZ}K_{k}^{T}. \end{array}$$

Step 7: Iterate from steps 2 to 6 until all samples are completed.

Under the condition of time-varying process-error and measurement noise covariance, we can infer that if $Q_{k-1}$ in (6) and $R_{k}$ in (9) could not be estimated timely, then inaccurate estimates would be made because, in (10), ${\hat{X}}_{k}$ is influenced by $K_{k}$ which is related to $R_{k}$ and ${\hat{X}}_{k/k-1}$.

### 2.2. Adaptive R Estimation

As proved by Li et al. in [27], for simplicity, assuming $Z_{1}(k)$ and $Z_{2}(k)$ are independent redundant measurements of a signal Z(k) from two sensors, they can be modeled as

(11) $$\left\{ \begin{array}{l} Z_{1}(k)=Z_{T}(k)+S_{1}(k)+V_{1}(k)\\ Z_{2}(k)=Z_{T}(k)+S_{2}(k)+V_{2}(k) \end{array}\right.$$

where $Z_{T}(k)$ denotes the true value of Z(k), $S_{1}(k)$ and $S_{2}(k)$ are steady items of the measurement errors, $V_{1}(k)$ and $V_{2}(k)$ are uncorrelated zero-mean random white noise.

The first-order-self-difference (FOSD) $\Delta Z_{1}$, $\Delta Z_{2}$ and the second-order-mutual-difference (SOMD) $\Delta Z_{12}$ are defined as

(12) $$\left\{ \begin{array}{l} \Delta Z_{1}(k)=Z_{1}(k)-Z_{1}(k-1)\\ \Delta Z_{2}(k)=Z_{2}(k)-Z_{2}(k-1)\\ \Delta Z_{12}(k)=\Delta Z_{1}(k)-\Delta Z_{2}(k) \end{array}\right.$$

Under the condition that the sampling interval is short enough, $S_{i}(k)-S_{i}(k-1)\approx 0,\;i=1,\;2$. The covariance of the random noise for measurement $Z_{1}(k)$ and $Z_{2}(k)$ can be estimated as

(13) $$\left\{ \begin{array}{l} R_{1}=\frac{E\left[\Delta Z_{12}(k)\Delta Z_{12}{\left(k\right)}^{T}\right]+E\left[\Delta Z_{1}(k)\Delta Z_{1}{\left(k\right)}^{T}\right]-E\left[\Delta Z_{2}(k)\Delta Z_{2}{\left(k\right)}^{T}\right]}{4}\\ R_{2}=\frac{E\left[\Delta Z_{12}(k)\Delta Z_{12}{\left(k\right)}^{T}\right]-E\left[\Delta Z_{1}(k)\Delta Z_{1}{\left(k\right)}^{T}\right]+E\left[\Delta Z_{2}(k)\Delta Z_{2}{\left(k\right)}^{T}\right]}{4} \end{array}\right.$$

The mathematical expectations in (13) are calculated as follows, because the statistical characteristics are stable over a relatively short period.

(14) $$\begin{array}{l} \left\{ \begin{array}{l} E\left[\Delta Z_{1}(k)\Delta Z_{1}{\left(k\right)}^{T}\right]\\ =\frac{1}{M}\sum_{m=0}^{M-1}\left\{\Delta Z_{1}\left(k-m\right)-E\left[\Delta Z_{1}\left(k\right)\right]\right\}{\left\{\Delta Z_{1}\left(k-m\right)-E\left[\Delta Z_{1}\left(k\right)\right]\right\}}^{T}\\ E\left[\Delta Z_{2}(k)\Delta Z_{2}{\left(k\right)}^{T}\right]\\ =\frac{1}{M}\sum_{m=0}^{M-1}\left\{\Delta Z_{2}\left(k-m\right)-E\left[\Delta Z_{2}\left(k\right)\right]\right\}{\left\{\Delta Z_{2}\left(k-m\right)-E\left[\Delta Z_{2}\left(k\right)\right]\right\}}^{T}\\ E\left[\Delta Z_{12}(k)\Delta Z_{12}{\left(k\right)}^{T}\right]\\ =\frac{1}{M}\sum_{m=0}^{M-1}\left\{\Delta Z_{12}(k-m)-E\left[\Delta Z_{12}\left(k\right)\right]\right\}{\left\{\Delta Z_{12}(k-m)-E\left[\Delta Z_{12}\left(k\right)\right]\right\}}^{T} \end{array}\right. \end{array}$$

where $E\left[\Delta Z_{i}\left(k\right)\right],\;i=1,\;2$ and $E\left[\Delta Z_{12}\left(k\right)\right]$ can be calculated by

(15) $$E\left[\Delta Z_{i}\left(k\right)\right]=\frac{1}{M}\sum_{m=0}^{M-1}\Delta Z_{i}\left(k-m\right)$$

(16) $$E\left[\Delta Z_{12}\left(k\right)\right]=\frac{1}{M}\sum_{m=0}^{M-1}\Delta Z_{12}\left(k-m\right)$$

where M is the sliding window width which can be empirically set to 30~60 [25].

In practice, in order to capture the transient behavior of the variation of $R_{k}$ in time while considering the smoothness, a fading memory calculation is implemented as [30]

(17) $$\left\{ \begin{array}{l} {\hat{R}}_{k}=\left(1-d_{k}\right){\hat{R}}_{k-1}+d_{k}R_{k}\\ d_{k}=\frac{1-b}{1-b^{k+1}},\;\left(0<b<1\right) \end{array}\right.$$

where b is the fading factor and in our work is set to 0.980, $R_{k}$ represents the direct output in (13) at time k, ${\hat{R}}_{k}$ is the final covariance matrix.

### 2.3. The Proposed Adaptive UKF Based on the Mahalanobis Distance of the Innovation Vector

As emphasized by many researchers [15,17,31], the parameters of the system model and the distribution of the measurement noise and the process noise could not be maintained as constants in practice all the time. In order to prevent the estimation from deteriorating or even diverging, caused by the model error, it is vital to determine and correct the mismatch between the real process error and the parameters’ preset. Herein, in our proposed method, based on hypothesis testing theory [14], the Mahalanobis distance of innovation vector is employed as a criterion to identify whether the system modeling error exists or not [17].

For the sake of simplicity, only one sensor of (1) is taken into consideration, thus, the superscript i is dropped. This paper develops a new method to improve the adaptability of the classical UKF against process model error based on the Mahalanobis distance. However, if the measurement noise matrix $R_{k}$ is also contaminated, then one single sensor cannot cope with this case. Thanks to the RMNCE, in MSIF, the $R_{k}$ can be estimated and is immune to the state estimation.

Define the innovation sequence $\varepsilon_{k}$ according to [15] as

(18) $$\varepsilon_{k}=Z_{k}-h\left({\hat{X}}_{k/k-1}\right)$$

Then $\varepsilon_{k}$ should be zero-mean Gaussian-distributed with covariance $E\left[\varepsilon_{k}\varepsilon_{k}^{T}\right]=H_{k/k-1}P_{k/k-1}H_{k/k-1}^{T}+{\hat{R}}_{k}$ [19], $H_{k/k-1}={\frac{\partial h}{\partial X}|}_{X={\hat{X}}_{{}_{k/k-1}}}$; the square of the Mahalanobis distance of the innovation should be $\chi^{2}$ distributed [4],

(19) $$M^{2}=\varepsilon_{k}^{T}\left\{E{\left[\varepsilon_{k}\varepsilon_{k}^{T}\right]}^{-1}\right\}\varepsilon_{k}∼\chi_{m}^{2}$$

where m is the degree of freedom.

Based on the hypothesis testing theory, let α be the given significance level, and then we have

(20) $$\mathrm{Pr}\left(M^{2}<\chi_{m,\alpha }^{2}\right)=1-\alpha \;\left(0\leq \alpha \leq 1\right)$$

where Pr(•) stands for the probability of a random event, $\chi_{m,\alpha }^{2}$ denotes the α-quantile of the distribution $\chi_{m}^{2}$.

If (20) does not hold, it can be deduced with high probability (1−α) that there exists process-error in the system (1), assuming that the observations are within reasonable bounds. Because there is more than one sensor in the system, (20) should be calculated for each sensor.

Different from [4,17], instead of a single parameter that acts on $P_{k/k-1}$

(21) $$P_{k/k-1}^{*}=\lambda_{k}P_{k/k-1}=\lambda_{k}\left(P_{XX}+\Gamma_{k-1}Q_{k-1}\Gamma_{k-1}^{T}\right)$$

We present a new algorithm that takes the advantage of sequences of both innovation and residual to correct the matrix Q directly by a diagonal matrix, and the procedure is as follows.

According to [15], the residual $\eta_{k}$ can be defined as

(22) $$\eta_{k}=Z_{k}-h\left({\hat{X}}_{k}\right)$$

According to [18], based on the principle of orthogonality [12], the residual sequences are uncorrelated from the measurements. So, the following equations should hold:

(23) $$E\left(\eta_{k}\eta_{k}^{T}\right)={\hat{R}}_{k}-H_{k}P_{k}H_{k}^{T}.$$

If $P_{k}$ is replaced by its definition in (10), then

(24) $$H_{k}P_{k}H_{k}^{T}=H_{k}\left(P_{k/k-1}-K_{k}P_{ZZ}K_{k}^{T}\right)H_{k}^{T}.$$

The traces of both sides should be equal:

(25) $$\begin{array}{ll} tr\left(\eta_{k}\eta_{k}{}^{T}\right) & =tr\left({\hat{R}}_{k}\right)-tr\left(H_{k}\left(P_{k/k-1}-K_{k}P_{ZZ}K_{k}^{T}\right)H_{k}^{T}\right)\\ & =tr\left({\hat{R}}_{k}\right)-tr\left(H_{k}\left(P_{k/k-1}^{*}+\Gamma_{k-1}Q\Gamma_{k-1}^{T}\right)H_{k}^{T}\right)+tr\left(H_{k}K_{k}P_{ZZ}K_{k}^{T}H_{k}^{T}\right) \end{array}$$

where $H_{k}={\frac{\partial h}{\partial X}|}_{X={\hat{X}}_{{}_{k}}}$, and $P_{k/k-1}^{*}$ is the predicted covariance without the additive process noise. If (23) does not hold, there will be some change in matrix Q. So, one scalar called the adaptive fading factor is generated based on the Equation (23) [16]:

(26) $$\begin{array}{l} P_{k/k-1}=P_{k/k-1}^{*}+\Lambda_{k}\Gamma_{k-1}Q_{k-1}\Gamma_{k-1}^{T}\\ \Lambda_{k}=\frac{tr\left({\hat{R}}_{k}\right)-tr\left(H_{k}P_{k/k-1}H_{k}^{T}\right)+tr\left(H_{k}K_{k}P_{ZZ}K_{k}^{T}H_{k}^{T}\right)-tr\left(\eta_{k}\eta_{k}{}^{T}\right)}{tr\left(H_{k}\Gamma_{k-1}Q\Gamma_{k-1}^{T}H_{k}^{T}\right)}. \end{array}$$

Instead of one scalar being calculated to tune the matrix Q, much higher accuracy could be obtained by using a matrix $\Lambda =diag\{\lambda_{1},\;\lambda_{2},\;\ldots ,\;\lambda_{m}\}$, in which m is the dimension of the Q.

So, an innovative adaptive method is proposed as

(27) $$P_{k}=P_{k/k-1}^{*}+\Gamma_{k-1}\Lambda_{k}Q_{k-1}\Gamma_{k-1}^{T},\Lambda_{k}=diag\{\lambda_{1},\;\lambda_{2},\;\ldots ,\;\lambda_{m}\}$$

(28) $$\Lambda_{k}=\frac{1}{2}{\left(H_{k}\Gamma_{k-1}\right)}^{-1}\left\{E\left[\varepsilon_{k}\varepsilon_{k}^{T}\right]-E\left[\eta_{k}\eta_{k}^{T}\right]+E\left[\left(\varepsilon_{k}-\eta_{k}\right){\left(\varepsilon_{k}-\eta_{k}\right)}^{T}\right]-2H_{k}P_{k/k-1}^{*}H_{k}^{T}\right\}{\left(\Gamma_{k-1}^{T}H_{k}^{T}\right)}^{-1}Q_{k-1}^{-1}.$$

To obtain (28), firstly, the relationship of $E\left[\left(\varepsilon_{k}-\eta_{k}\right){\left(\varepsilon_{k}-\eta_{k}\right)}^{T}\right]$, $E\left[\varepsilon_{k}\varepsilon_{k}^{T}\right]$ and $E\left[\eta_{k}\eta_{k}^{T}\right]$ are given; then the matrix $\Lambda_{k}$ is solved but not in the form of the trace of a matrix.

The covariance of innovation is:

(29) $$E\left[\varepsilon_{k}\varepsilon_{k}^{T}\right]=H_{k}P_{k/k-1}H_{k}^{T}+{\hat{R}}_{k}.$$

The covariance of difference sequences between innovation and residual is

(30) $$E\left[\left(\varepsilon_{k}-\eta_{k}\right){\left(\varepsilon_{k}-\eta_{k}\right)}^{T}\right]=E\left[\varepsilon_{k}\varepsilon_{k}^{T}\right]+E\left[\eta_{k}\eta_{k}^{T}\right]-E\left[\varepsilon_{k}\eta_{k}^{T}\right]-E\left[\eta_{k}\varepsilon_{k}^{T}\right].$$

A deep look should be taken at $\varepsilon_{k}$ and $\eta_{k}$ before calculating (30)

(31) $$\begin{array}{ll} \varepsilon_{k} & =Z_{k}-h\left({\hat{X}}_{k/k-1}\right)=h\left(X_{k}\right)+V_{k}-h\left({\hat{X}}_{k/k-1}\right)\\ & \approx H_{k/k-1}\left(X_{k}-{\hat{X}}_{k/k-1}\right)+V_{k}\\ & =H_{k/k-1}{\tilde{X}}_{k/k-1}+V_{k}\\ \eta_{k} & =Z_{k}-h\left({\hat{X}}_{k}\right)=h\left(X_{k}\right)+V_{k}-h\left({\hat{X}}_{k}\right)\\ & \approx H_{k}\left(X_{k}-{\hat{X}}_{k}\right)+V_{k}\\ & =H_{k}{\tilde{X}}_{k}+V_{k} \end{array}$$

where ${\tilde{X}}_{k/k-1}$ and ${\tilde{X}}_{k}$ represent the prediction error and the estimation error, respectively.

The first two items in the right side of (30) can be derived based on (31) as

(32) $$\begin{array}{ll} E\left[\varepsilon_{k}\varepsilon_{k}^{T}\right] & =E\left[\left(H_{k/k-1}{\tilde{X}}_{k/k-1}+V_{k}\right){\left(H_{k/k-1}{\tilde{X}}_{k/k-1}+V_{k}\right)}^{T}\right]\\ & =H_{k/k-1}P_{k/k-1}H_{k/k-1}^{T}+{\hat{R}}_{k}\\ E\left[\eta_{k}\eta_{k}^{T}\right] & =E\left[\left(H_{k}\left(X_{k}-{\hat{X}}_{k}\right)+V_{k}\right){\left(H_{k}\left(X_{k}-{\hat{X}}_{k}\right)+V_{k}\right)}^{T}\right]\\ & ={\hat{R}}_{k}-H_{k}P_{k}H_{k}^{T}. \end{array}$$

Because the sample frequency is high, the Jacobian matrices $H_{k/k-1}\approx H_{k}$, and

$E\left[\varepsilon_{k}\eta_{k}^{T}\right]\approx E\left[\eta_{k}\varepsilon_{k}^{T}\right]$, Ge et al. gave the proof in [15]

(33) $$\begin{array}{ll} E\left[\varepsilon_{k}\eta_{k}^{T}\right] & =E\left[\left(H_{k/k-1}\left({\tilde{X}}_{k/k-1}\right)+V_{k}\right){\left(H_{k/k-1}\left({\tilde{X}}_{k}\right)+V_{k}\right)}^{T}\right]\\ & =H_{k/k-1}P_{k}H_{k}^{T}+{\hat{R}}_{k}\left(I-K_{k}H_{k}^{T}\right). \end{array}$$

Then, based on (32) and (33), namely,

(34) $$\begin{array}{ll} E\left[\left(\varepsilon_{k}-\eta_{k}\right){\left(\varepsilon_{k}-\eta_{k}\right)}^{T}\right] & =E\left[\varepsilon_{k}\varepsilon_{k}^{T}\right]+E\left[\eta_{k}\eta_{k}^{T}\right]-E\left[\varepsilon_{k}\eta_{k}^{T}\right]-E\left[\eta_{k}\varepsilon_{k}^{T}\right]\\ & \approx H_{k/k-1}P_{k/k-1}H_{k/k-1}^{T}+{\hat{R}}_{k}+{\hat{R}}_{k}-H_{k}P_{k}H_{k}^{T}\\ & \;-2\left(H_{k/k-1}P_{k}H_{k}^{T}-{\hat{R}}_{k}K_{k}H_{k}^{T}+{\hat{R}}_{k}\right)\\ & =H_{k/k-1}P_{k/k-1}H_{k/k-1}^{T}-H_{k}P_{k}H_{k}^{T}\\ & \approx H_{k}P_{k/k-1}H_{k}^{T}-H_{k}P_{k}H_{k}^{T}\\ & =H_{k}\left(P_{k/k-1}-P_{k}\right)H_{k}^{T}\\ & =H_{k}K_{k}P_{ZZ}K_{k}^{T}H_{k}^{T} \end{array}$$

According to (29), replacing ${\hat{R}}_{k}$ by $E\left[\varepsilon_{k}\varepsilon_{k}^{T}\right]-H_{k}P_{k/k-1}H_{k}^{T}$ in (25) can be rewritten without trace calculation based on (32), (33), and $P_{k/k-1}=P_{k/k-1}^{*}+\Gamma_{k-1}\Lambda_{k}Q_{k-1}\Gamma_{k-1}^{T}$

(35) $$\begin{array}{ll} \;E\left[\eta_{k}\eta_{k}{}^{T}\right] & =E\left[\varepsilon_{k}\varepsilon_{k}^{T}\right]-H_{k}P_{k/k-1}H_{k}^{T}-H_{k}P_{k/k-1}H_{k}^{T}+H_{k}K_{k}P_{vv}K_{k}^{T}H_{k}^{T}\\ & =E\left[\varepsilon_{k}\varepsilon_{k}^{T}\right]-2H_{k}P_{k/k-1}H_{k}^{T}+H_{k}K_{k}P_{ZZ}K_{k}^{T}H_{k}^{T}\\ & =E\left[\varepsilon_{k}\varepsilon_{k}^{T}\right]-2H_{k}\left(P_{k/k-1}^{*}+\Lambda_{k}\Gamma_{k-1}Q_{k-1}\Gamma_{k-1}^{T}\right)H_{k}^{T}+E\left[\left(\varepsilon_{k}-\eta_{k}\right){\left(\varepsilon_{k}-\eta_{k}\right)}^{T}\right]. \end{array}$$

Hence, (28) is obtained by rearranging (35). Normally, the covariance equations stacked above are calculated as

(36) $$\begin{array}{l} \;E\left[\varepsilon_{k}\varepsilon_{k}^{T}\right]=\frac{1}{M}\sum_{m=0}^{M-1}\left(\varepsilon_{k-m}-\bar{\varepsilon }\right){\left(\varepsilon_{k-m}-\bar{\varepsilon }\right)}^{T}\\ E\left[\eta_{k}\eta_{k}{}^{T}\right]=\frac{1}{M}\sum_{m=0}^{M-1}\left(\eta_{k-m}-\bar{\eta }\right){\left(\eta_{k-m}-\bar{\eta }\right)}^{T}\\ E\left[\left(\varepsilon_{k}-\eta_{k}\right){\left(\varepsilon_{k}-\eta_{k}\right)}^{T}\right]=\frac{1}{M}\sum_{m=0}^{M-1}\left(\varepsilon_{k-m}-\eta_{k-m}\right){\left(\varepsilon_{k-m}-\eta_{k-m}\right)}^{T} \end{array}$$

in which M is the windows size, $\bar{\varepsilon }=\frac{1}{M}\sum_{m=0}^{M-1}\varepsilon_{k-m}$, and $\bar{\eta }=\frac{1}{M}\sum_{m=0}^{M-1}\eta_{k-m}$.

**Remark** **1.** *In Equation (28), the inverse matrices of* ${\left(H_{k}\Gamma_{k-1}\right)}^{-1}$ *and* $Q_{k-1}^{-1}$ *are required to be calculated. In practice,* $Q_{k-1}$ *is normally a positive definite diagonal matrix, but* $H_{k}\Gamma_{k-1}$ *may be not an invertible matrix, or not even a square matrix. Considering that* $\Lambda_{k}$ *is a diagonal matrix, we can obtain the elements by solving the following equation:*

(37) $$H_{k}\Gamma_{k-1}\Lambda_{k}Q_{k-1}^{}\Gamma_{k-1}^{T}H_{k}^{T}=\frac{1}{2}\left\{E\left[\varepsilon_{k}\varepsilon_{k}^{T}\right]-E\left[\eta_{k}\eta_{k}^{T}\right]+E\left[\left(\varepsilon_{k}-\eta_{k}\right){\left(\varepsilon_{k}-\eta_{k}\right)}^{T}\right]-2H_{k}P_{k/k-1}^{*}H_{k}^{T}\right\}.$$

**Remark** **2.** *In Equation (37),*$H_{k}$*and*$H_{k}^{T}$*are the Jacobian matrices. They normally can be considered as the slope at*${\hat{X}}_{k}$*. During the course of numerical calculation,*$H_{k}$*and*$H_{k}^{T}$*may be very small, so one or more elements of*$\Lambda_{k}$*will be large enough to cause*$P_{k}$*to be a negative definite in the next step. To solve this problem, a threshold value is used to limit the element of*$\Lambda_{k}$*. Meanwhile, if*$\lambda_{i}$*is not positive in numerical applications, it is always reset to the absolute value of its estimate[28]*.

**Remark** **3.** *In (28) or (37), the result of the first three items in the right side of equations may be much greater than the last item, because the assumption that the innovation and residual sequences are orthogonal is statistically ideal. In other words, for a limited set of the sample, (36) is commonly biased [28]. However, in many real-time or online systems, the sliding window could not be set very large to meet the assumption. So, the tradeoff should be made between the numerical stability and real-time performance.*

### 2.4. The Decentralized MSIF

Due to the expensive computational cost for high-dimension matrices and low stability when some measurements are abnormal [10], i.e., one or more sensors provide information with large noises, or just noises, the centralized architecture is not adopted widely. This work adopts the decentralized MSIF structure similar to [1], which is derived from [32]. For simplicity, the time instant k in $X_{k}$ is dropped in this section [1].

Let ${\hat{X}}^{i},i=1,2,\ldots ,l$ be unbiased estimators of X in (1) and the estimation errors be ${\tilde{X}}^{i}=X-{\hat{X}}^{i},i=1,2,\ldots ,l$. Assume that $X^{i}$ and $X^{j}$ are correlated, the covariance and cross covariance are $P^{ii}$ and $P^{ij}$, respectively. The optimal fusion estimator ${\hat{X}}_{op}$ with matrix weights can be described as

(38) $${\hat{X}}_{op}=\sum_{i=1}^{l}{\bar{A}}^{i}{\hat{X}}^{i}={\bar{A}}^{1}{\hat{X}}^{1}+{\bar{A}}^{2}{\hat{X}}^{2}+\ldots +{\bar{A}}^{l}{\hat{X}}^{l}$$

where the optimal matrix weights ${\bar{A}}^{i},\;i=1,\;2,\;\ldots ,\;l$ are to be determined.

The globally optimal information fusion Kalman filter ${\hat{X}}_{op}$ of the state X, based on the principle of linear minimum variance, will satisfy the following conditions [1,17]:

**Condition** **1.** ${\hat{X}}_{op}$*must be the unbiased estimation of*X*,namely,* $E\left[{\hat{X}}_{op}\right]=E\left[X\right]$*.*

**Condition** **2.** ${\hat{X}}_{op}$ *makes* tr{P} *minimum, in which* P *is the error covariance matrix of* ${\hat{X}}_{op}$ *.*

For simplicity, we denote $A={\left[A^{1},\;A^{2},\;\ldots ,\;A^{l}\right]}^{T}$. Our aim is to find A to construct the unbiased estimator

(39) $$\hat{X}=\sum_{i=1}^{l}A^{i}{\hat{X}}^{i}=A^{1}{\hat{X}}^{1}+A^{2}{\hat{X}}^{2}+\ldots +A^{l}{\hat{X}}^{l}$$

where $A^{i},\;i=1,\;2,\;\ldots ,\;l$ are arbitrary matrices.

If ${\hat{X}}_{op}$ is an unbiased estimator for X, the following condition should be fulfilled,

(40) $$E\left[{\hat{X}}_{op}\right]=E\left[X\right]$$

Taking the expectation of both sides of (38) yields,

(41) $$E\left[{\hat{X}}_{op}\right]=E\left[\sum_{i=1}^{l}A^{i}{\hat{X}}^{i}\right]=\sum_{i=1}^{l}A^{i}E\left[{\hat{X}}^{i}\right].$$

So (43) is obtained

(42) $$I_{n}=\sum_{i=1}^{l}A^{i}=A^{1}+A^{2}+\cdots +A^{l}.$$

Based on (38) and (42), we get the fusion estimation error

(43) $$\tilde{X}=X-\sum_{i=1}^{l}A^{i}{\hat{X}}^{i}=\sum_{i=1}^{l}A^{i}(X-{\hat{X}}^{i})=\sum_{i=1}^{l}A^{i}{\tilde{X}}^{i}.$$

The error variance matrix of the fusion estimator is

(44) $$P=E\left[\tilde{X}{\tilde{X}}^{T}\right]=A^{T}\Sigma A$$

where $\Sigma =\left(P_{ij}\right)\;i,\;j=1,\;2,\;\ldots ,\;l$ is a symmetric positive definite matrix.

Now the problem is converted to a classic one: under the constraint of (42), to solve the minimum of $tr\{P\}=tr\{A^{T}\Sigma A\}$ by applying the Lagrange multiplier

(45) $$F=tr\{P\}+tr\{\Lambda [A^{T}E-I_{n}]\}$$

where $\Lambda \in {\mathbb{R}}^{n\times n}$ is the Lagrange multiplier, $E={\left[I_{n},\;I_{n},\;\ldots ,\;I_{n}\right]}^{T}\in {\mathbb{R}}^{nN\times n}$; and $I_{n}$ represents an n-dimensional identity matrix.

By setting $\partial F/{\partial A|}_{A=\bar{A}}=0$, and noting that $\Sigma^{T}=\Sigma$, we have

(46) $$\Sigma \bar{A}+E\Lambda =0$$

By substituting (42) into (46)

(47) $$\left(\begin{array}{cc} \Sigma & E\\ E^{T} & 0 \end{array}\right)\left(\begin{array}{c} \bar{A}\\ \Lambda \end{array}\right)=\left(\begin{array}{l} 0\\ I_{n} \end{array}\right)$$

Because Σ is a symmetric positive definite matrix, $E^{T}\Sigma^{-1}E$ is nonsingular. Based on the matrix theory, (47) can be solved

(48) $$\left(\begin{array}{c} \bar{A}\\ \Lambda \end{array}\right)={\left(\begin{array}{cc} \Sigma & E\\ E^{T} & 0 \end{array}\right)}^{-1}\left(\begin{array}{c} 0\\ I_{n} \end{array}\right)=\left(\begin{array}{c} \Sigma^{-1}E{\left(E^{T}\Sigma^{-1}E\right)}^{-1}\\ -{\left(E^{T}\Sigma^{-1}E\right)}^{-1} \end{array}\right)$$

From (48), the matrix Σ should be calculated to obtain the global optimal state estimation. The diagonal elements $P^{ii},\;i=1,\;2,\;\ldots ,\;l$ in the matrix Σ can be directly calculated by the error covariance matrix of the state estimation in the ith local filter. However, the cross-covariance matrix is difficult to get. In our work, we use the result given by Gao et al. base on UT [17].

(49) $$\begin{array}{ll} P_{k}^{ij} & =\sum_{s=0}^{2n}\omega_{s}^{\left(c\right)}\left(\chi_{s,k/k-1}^{\left(i\right)}-{\hat{X}}_{s,k/k-1}^{\left(i\right)}\right){\left(\chi_{s,k/k-1}^{\left(j\right)}-{\hat{X}}_{s,k/k-1}^{\left(j\right)}\right)}^{T}\\ & \;-\left[\sum_{s=0}^{2n}\omega_{s}^{\left(c\right)}\left(\chi_{s,k/k-1}^{\left(i\right)}-{\hat{X}}_{s,k/k-1}^{\left(i\right)}\right){\left(\gamma_{s,k/k-1}^{\left(j\right)}-{\hat{Z}}_{s,k/k-1}^{\left(j\right)}\right)}^{T}\right]{\left(K^{\left(j\right)}\right)}^{T}\\ & \;-K^{\left(i\right)}\left[\sum_{s=0}^{2n}\omega_{s}^{\left(c\right)}\left(\gamma_{s,k/k-1}^{\left(i\right)}-{\hat{Z}}_{s,k/k-1}^{\left(i\right)}\right){\left(\chi_{s,k/k-1}^{\left(j\right)}-{\hat{X}}_{s,k/k-1}^{\left(j\right)}\right)}^{T}\right]\\ & \;+K^{\left(i\right)}\left[{\sum_{s=0}^{2n}\omega_{s}^{\left(c\right)}\left(\gamma_{s,k/k-1}^{\left(i\right)}-{\hat{Z}}_{s,k/k-1}^{\left(i\right)}\right)\left(\gamma_{s,k/k-1}^{\left(j\right)}-{\hat{Z}}_{s,k/k-1}^{\left(j\right)}\right)}^{T}\right]{\left(K^{\left(j\right)}\right)}^{T} \end{array}$$

where $\chi_{s,k/k-1}^{\left(i\right)}$ is the sigma point transformed by the nonlinear function f(•) in (6) for the ith sensor; $\gamma_{s,k/k-1}^{\left(j\right)}$ represents the sigma points transformed by the nonlinear function h(•) in (8); and s=0, 1, …, 2n denotes the order of the transformed sigma points.

**Remark** **4.** *In order to maintain* $P_{k}^{ij}$ *as a positive definite, some exceptions should be taken into consideration. Firstly, Equation (42) should be examined immediately when* Σ *is fulfilled by* $P_{k}^{ij}$ *. This step is necessary because there may be truncation errors during calculation. If the constraint defined by (42) is not satisfied, set* $P_{k}^{ij}=P_{k-1}^{ij}$ *.*

Secondly, in MSIF, the optimal estimation ${\hat{X}}_{op}$ theoretically lies in the closed interval:

(50) $$min\left(norm2({\hat{X}}^{i})\right)\leq norm2\left({\hat{X}}_{op}\right)\leq max\left(norm2\left({\hat{X}}^{i}\right)\right),\;i=1,\;2,\;\ldots ,\;l$$

where norm2(•) denotes the 2-norm. If (50) is not maintained, the following degenerative methods (52) or (53) can be used:

(51) $$P_{k}^{ij}(row,col)=0.\;when\;row\neq col$$

(52) $${\hat{X}}_{op}=\frac{1}{l}\sum_{i=1}^{l}{\hat{X}}_{}^{i}.$$

## 3. The Proposed Method with Both Adaptivity and Robustness

In Section 2, we proposed the innovative algorithm to adapt Q when process-error exists. In this section, based on the hypothesis theory, the adopted judging criterion on process-error detection is given [4,17]. However, the drawbacks of the methods proposed by [4] and [17] are that the Q and R could not be estimated at the same time. Severe problems would be caused under the dilemma to decide which one should be adapted: Q, R, or both? To conquer this challenge, the RMNCE is employed to estimate the noise covariance of each sensor, then a decision is made whether the matrix R is suffering gross errors or not. Based on RMNCE, the decision is made easier by MSIF because the estimations of R are obtained with relatively high accuracy.

Briefly, in our proposed MSIF architecture, the matrix R of each sensor is estimated by RMNCE, and is denoted as ${\hat{R}}_{k}^{i}$; meanwhile, the process-error can be corrected by adapting Q if necessary. For the sake of simplicity, only one sensor of (1) is taken into consideration, so the superscript i in ${\hat{R}}_{k}^{i}$ could be dropped.

### 3.1. Robust R Estimaiton Based on RMNCE

As described in Section 2.2, the main advantage of RMNCE is that the estimate of variance is based only on measurements and hence can be immune to the state estimation error [27]. So, ${\hat{R}}_{k}$ calculated by (17) can be considered as the benchmark to test whether the process error exists or not. Let $R_{in}$ be calculated by the following equation [28]

(53) $$R_{in}=E\left[\varepsilon_{k}\varepsilon_{k}^{T}\right]-H_{k}P_{k/k-1}H_{k}^{T}.$$

The difference between $R_{in}$ and ${\hat{R}}_{k}$ could be large if process error exists. The quotient is used as an indicator to detect the process error as

(54) $$indicator=\frac{tr\left({\hat{R}}_{k}\right)}{tr\left(R_{in}\right)}=\left\{ \begin{array}{l} {}[0.90,\;1.10]:normal\\ esle:process\;erorr\;\mathrm{may}\;\mathrm{exist} \end{array}\right.$$

where $tr\left({\hat{R}}_{k}\right)$ and $tr\left(R_{in}\right)$ denote the trace of ${\hat{R}}_{k}$ and $R_{in}$, respectively.

When the quotient is around 1.0, it could be deduced with great probability that there is no process error. In this paper, a closed interval from 0.90 to 1.10 is used as the normal range. Otherwise, if the indicator is larger than 1.10 or smaller than 0.90, there is process error with great probability.

**Remark** **5.** *If the diagonal elements differ by more than one order of magnitude, it is better to calculate the indicator separately. For example, the matrices ${\hat{R}}_{k}$* *and $R_{in}$* *are as*

(55) $${\hat{R}}_{k}=\left[\begin{array}{cc} 10 & 0\\ 0 & 0.1 \end{array}\right],R_{in}=\left[\begin{array}{cc} 10 & 0\\ 0 & 1 \end{array}\right].$$

Although the indicator calculated by (54) belongs to the closed interval, $\frac{tr\left({\hat{R}}_{k}\right)}{tr\left(R_{in}\right)}=\frac{10.01}{11}=0.918\in [0.90,\;1.10]$, it is obvious that the second element in the diagonal differs 10 times in $R_{in}$ than ${\hat{R}}_{k}$.

**Remark** **6.** *The sliding window width for calculating* $R_{in}$ *and* ${\hat{R}}_{k}$ *should be the same. In this paper, it is set to 50.*

In our MSIF architecture, we also proposed the following matrix weights ${\bar{A}}_{R^{i}}$ as an alternative to (48).

Consider a MSIF system with three sensors, the noise covariance matrices ${\hat{R}}_{i},\;i=1,\;2,\;3$ could be obtained by (13). Because the noise covariance matrices are usually diagonal, the following equation is employed to calculate the ${\bar{A}}_{R^{i}}$

(56) $${\bar{A}}_{R^{i}}(j,j)=diag\{\frac{1}{{\hat{R}}^{i}\left(j,j\right)}\times \left(\sum_{l=1}^{3}\frac{1}{{\hat{R}}^{l}\left(j,j\right)}\right)\},\;j=1,\;2,\;\ldots ,\;m.$$

where m is the dimension of $Z^{i}$.

**Remark** **7.** *In our proposed MSIF structure, all the sensors have the same measurement model, so all the* ${\bar{A}}_{R^{i}}$ *have the same dimension. If a MSIF system has different sensors, the variation of (56) should be derived specifically. However, the core idea is that the smaller* ${\hat{R}}^{i}\left(j,\;j\right)$ *is, the greater weight* ${\bar{A}}_{R^{i}}(j,\;j)$ *is. A degenerated case is that if one or more elements of* X *are observed only by one sensor, assuming sensor* i *, then*

(57) $${\bar{A}}_{R^{i}}=\left[\begin{array}{cc} I^{r} & 0^{r}\\ 0^{m-r} & {\bar{A}}_{R^{i}}^{m-r} \end{array}\right],{\bar{A}}_{R^{j}}=\left[\begin{array}{cc} 0^{r} & 0^{r}\\ 0^{m-r} & {\bar{A}}_{R^{j}} \end{array}\right],j\neq i$$

*where* r *denotes the number of elements in* X *that are only observed by sensor* i *. Hence, this is not a MSIF anymore, and this paper would not discuss this case any further.*

**Remark** **8.** *When there are* l≥2 *sensors in a MSIF system, there are basically two options to utilize (13):*

**Option** **1.** *To calculate two matrices* ${\hat{R}}_{k}$ *one time, so (13) and the relative equations will be run* l−2 *times at least.*

**Option** **2.** *To calculate all matrices* ${\hat{R}}_{k}$ *one time, then the classical least square method is used to solve the overdetermined equations, which are derived by the variation of (13). In this paper, Option 1 is adopted.*

### 3.2. The Adaptive and Robust UKF Algorithm for MSIF (ARUKF-MSIF)

In this subsection, the complete scheme is given. The proposed Algorithm 1 ARUKF-MSIF aimed at target tracking can be implemented as follows.
**Algorithm 1.** ARUKF-MSIF algorithmInitiation: initiate the l$\;\mathrm{sensors}’\;\mathrm{filters}\;\mathrm{with}\;{\hat{X}}^{i}{}_{0}$$,\;P_{0}^{ii}$$,\;P_{0}^{ij}$, α$,\;\mathrm{and}\;\chi_{m,\alpha }^{2}$;Step 1: State prediction through (5)–(7) for each sensor.Step 2: Observation prediction through (7) and (8) for each sensor.
Step 3: Estimate matrix $R^{i}$ for each sensor through (12) to (17).Step 4: Process-error judgment through (20) and (54).Step 5: Abnormal innovation distinguishing.        5.1 If (20) and (54) holds:                   5.1.1 Go to step 6.        5.2 Else:                   5.2.1 Adapt Q through (28).Step 6: Calculate Kalman gain and filtering through (9) and (10).Step 7: MSIF implementation.        7.1 Calculate $P_{}^{ij}$ through (49).        7.2 Calculate matrix weights $\bar{A}$ through (48) or (56), generate the optimal estimation ${\hat{X}}_{op}$.Step 8: For the next iteration, repeat steps from 1 to 7.

**Figure 1:**
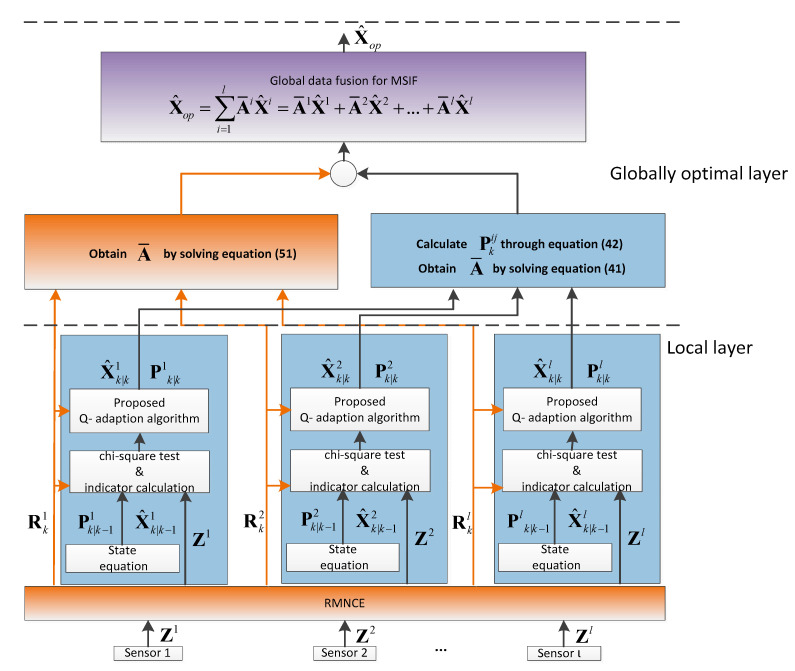
The framework of the proposed ARUKF-MSIF method.

The framework of the proposed ARUKF-MSIF methodology is shown in Figure 1. It has a two-layer fusion structure, the local layer and the globally optimal layer.

Based on RMNCE, the measurement noise covariance of each sensor is estimated. Every sensor estimates the states independently in the local layer. If any sensor subsystem detects the process-error by the chi-square test or the indicator proposed by this paper suggests the existence of the process-error, then the proposed Q-adaption algorithm is employed to correct this mismatch. The globally optimal layer is the final fusion center, where the optimal matrix weights are determined.

## 4. Simulations and Discussion

In this section, a set of numerical simulations of the radar tracking problems will be presented to illustrate the effectiveness of the proposed ARUKF-MSIF.

### 4.1. Process Model and Measurement Model

Similar to [1], consider the radar tracking system with three sensors. The differences between our model and [1] are threefold.

Firstly, our model is more complicated and challenging: a two-dimension trajectory with time-varying process-error and/or measurement noise. In order to illustrate the effectiveness of our proposed method, the uncertainty of the system contains not only the measurement outliers/failures argued in [1] but also the process-error as in [15], which is usually the case in practice.

Secondly, all the sensors have the same measurement model in our simulation. With the development of hardware technology, more and more systems can afford redundant equipment to apply the multi-sensor information fusion into practical projects because the price of hardware is getting cheaper and cheaper. As proved by [6], if the sensors in decentralized structure have identical measurement matrices as in the centralized one, the two fusion methods are functionally equivalent. By taking advantage of the much smaller computational burden of the decentralized structure and the same accuracy as the centralized one, the simulation is arranged with three identical sensors whose noise characteristics are different.

**Figure 2:**
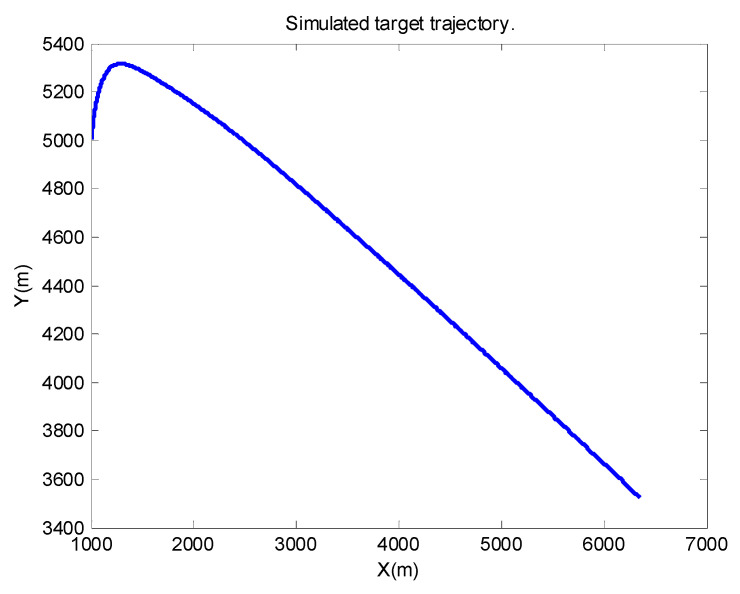
Flight trajectory of the target.

**Figure 3:**
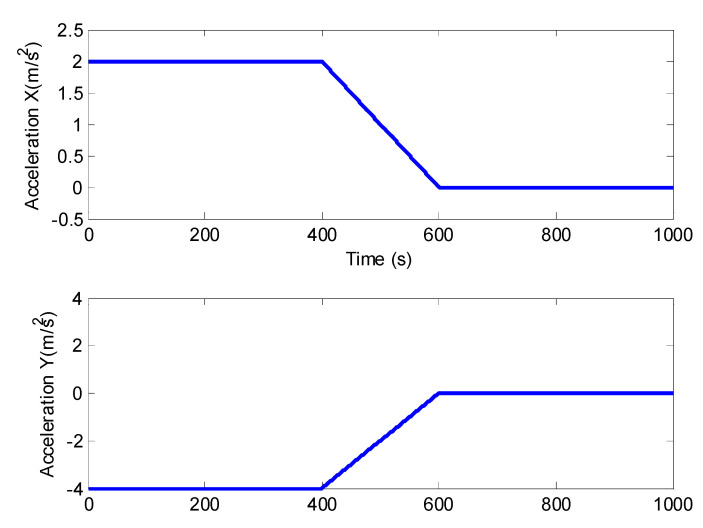
True acceleration during the trajectory.

Thirdly, the 2-D motion is taken into consideration as with [15,31]. The function of the radar can be demonstrated well enough; meanwhile, the model is not so complicated as the 3-D one. The simulated target trajectory is depicted in (Figure 2), and the true acceleration is shown in (Figure 3).

Consider that a target trajectory is in x–y plane. The position, velocity, and acceleration of the objective at time k are represented by the vector $X_{k}=\left[x_{k},\;y_{k},\;{\dot{x}}_{k},\;{\dot{y}}_{k},\;{\ddot{x}}_{k},\;{\ddot{y}}_{k}\right]$ in the Cartesian coordinates. The dynamic equation for the target movement is as [15,31],

(58) $$X_{k}=\left[\begin{array}{cccccc} 1 & 0 & T & 0 & T^{2}/2 & 0\\ 0 & 1 & 0 & T & 0 & T^{2}/2\\ 0 & 0 & 1 & 0 & T & 0\\ 0 & 0 & 0 & 1 & 0 & T\\ 0 & 0 & 0 & 0 & 1 & 0\\ 0 & 0 & 0 & 0 & 0 & 1 \end{array}\right]X_{k-1}+\Gamma_{k-1}W_{k-1},\;\;\;\;\Gamma_{k-1}=\left[\begin{array}{cc} T^{2}/2 & 0\\ 0 & T^{2}/2\\ T & 0\\ 0 & T\\ 1 & 0\\ 0 & 1 \end{array}\right]$$

where T denotes the sampling period.

The initial state is $X_{0}=\left[1000\;m,\;5000\;m,\;10\;m/s,\;50\;m/s,\;2\;m/s^{2},-4\;m/s^{2}\right]$, the process noise covariance matrix is

(59) $$Q_{k-1}=\left[\begin{array}{cc} 0.001 & 0\\ 0 & 0.001 \end{array}\right]$$

The multi-sensor observation systems are composed by three radars with the same measurement model described as

(60) $$Z_{k}^{i}=\left[\begin{array}{l} r_{k}^{i}\\ \varphi_{k}^{i} \end{array}\right]=\left[\begin{array}{l} \sqrt{x_{k}^{2}+y_{k}^{2}}\\ \arctan\left(\frac{y_{k}}{x_{k}}\right) \end{array}\right]+V_{k}^{i},\;i=1,\;2,\;3$$

where $r_{k}^{i}$ and $\varphi_{k}^{i}$ denote the slant range and azimuth angle in polar coordinates measured by the ith radar, respectively. The variance matrices of $V_{k}^{i},\;i=1,\;2,\;3$ are different from each other.

### 4.2. Simulations

To demonstrate the effectiveness of our proposed method, the following three cases are designed specifically.

**Case** **1:** *The noise covariance matrices* $R^{i}=diag\left[i^{2}\times 100,\;i^{2}\times 0.01\right],\;i=1,\;2,\;3$ *are known and the process noise covariance matrix* Q *varies over time. The process noise covariance matrix is assigned to be* Q=diag[0.015, 0.015] *during the epochs [200, 400].*

**Case** **2:** *The measurement noise covariance matrices* $R^{i}$ *are uncertain, and the process noise covariance matrix* Q=diag[0.001, 0.001] *is known. The measurement noise covariance matrix* $R^{1}$ *is assigned to be* $R^{1}=10\times diag\left[1^{2}\times 100,\;1^{2}\times 0.01\right]$ *during the epochs [200, 400], and it is set to the same value as in Case 1 for the remaining periods.*

**Case** **3:** *Not only the measurement noise covariance matrices* $R^{1}$ *but also the process noise covariance matrix* Q *are uncertain. The changes in Case 1 and Case 2 are implemented simultaneously in this case.*

Different from [15], in which the dynamic maneuver is simulated during a period when both the $R^{i}$ and the Q stay unchanged, in our simulations, all cases are designed to set the interval of the maneuver to the same one when $R^{1}$ and/or Q varies. So, the situations in our simulations are much harsher than the ones in [15]. The interacting multiple model (IMM) is employed by Ge et al. [15] to compare the algorithms; however, in this paper, we do not take the IMM method into consideration because its computational load is higher than the algorithm proposed by [15], and the complexity of our algorithm to adapt the Q is similar to [15]. Moreover, the Markov transition matrix used in IMM is obtained on the basis of statistics on the system evolution [33], so in order to employ the IMM, the transition matrix should be available. However, it could not be obtained precisely in practice.

For Case 1, the reason why only the matrix Q is uncertain is that we want to illustrate the effectiveness of our proposed method contrast to several algorithms which focus on the Q adaption. Except for the standard UKF, the AUKF algorithm proposed by [19], the adaptive fading UKF (AFUKF) method developed by [18], and the N-UKF given by [32] are taken into consideration together with ours to run the simulation.

For Case 2, to test the validity of our proposed MSIF structure with RMNCE, only $R^{1}$ changes during the specified intervals. The algorithms contained in the simulation are the improved Sage–Husa adaptive method in [26], the N-UKF, the standard UKF, and ours.

It is natural for Case 3 that the algorithms with both adaptability and robustness claimed by their inventors are taken into consideration except for the standard UKF and the improved Sage–Husa adaptive method. So, the N-UKF, the robust adaptive UKF proposed by [34], and our proposed method are applied to track the target.

For each case, $R^{1}$ of the sensor 1 is used by every single method. From 50 times of the Monte Carlo simulation in all cases, the root mean square error (RMSE) of each moment is obtained as

(61) $$RMSE_{k}=\sqrt{\frac{1}{N}\sum_{i=1}^{N}\left[{\left({\hat{x}}_{k}^{i}-x_{k}\right)}^{2}+{\left({\hat{y}}_{k}^{i}-y_{k}\right)}^{2}\right]}$$

where  is the total times of simulation, $\left({\hat{x}}_{k}^{i},\;{\hat{y}}_{k}^{i}\right)$ represents the filtering position of the target at time instant k in the ith simulation.

**Figure 4:**
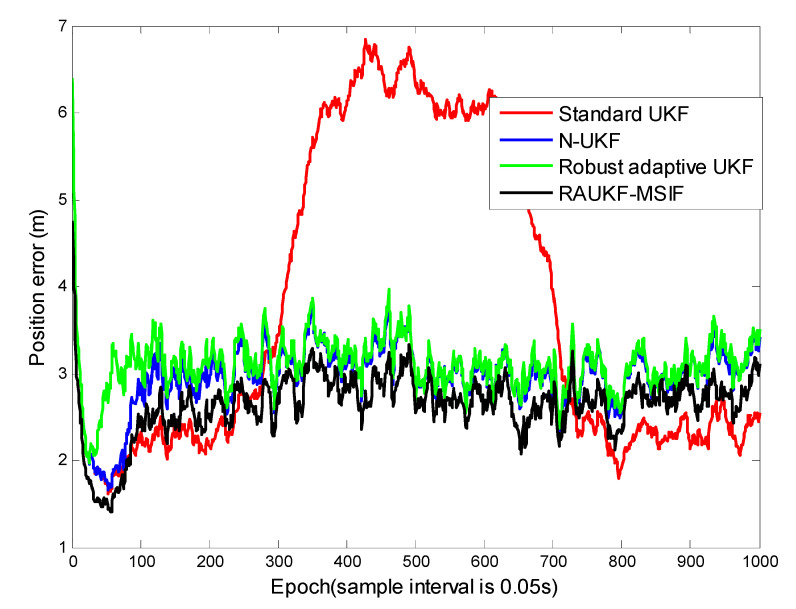
Position tracking errors for Case 1.

For the first case, the position tracking errors of the standard UKF, adaptive fading UKF, N-UKF, and our proposed method are shown in (Figure 4). To illustrate the performance of these algorithms clearly, we list the means and variance of the position tracking errors during the epochs [200, 600] and [601, 1000].

The epochs of Case 1 can be divided into three intervals: [0, 199], [200, 600], and [601, 1000]. The positioning errors during the first interval are close to each other because there is not process-modeling error. In the second interval, the maneuver of the target deteriorates the performance of the standard UKF. For the robust adaptive UKF, a modulation is made to prevent the algorithm divergence, i.e., a limitation is used for the adaptive factor. It can be seen clearly that all the methods except for the standard UKF can maintain their accuracy and robustness due to the Q adaption strategy used. From Table 1, the conclusion can be made that our proposed algorithm can resist the influence from both the time-varying process and the maneuvering motion models. The positioning errors estimated by our method remained the lowest among all the algorithms.

**Figure 5:**
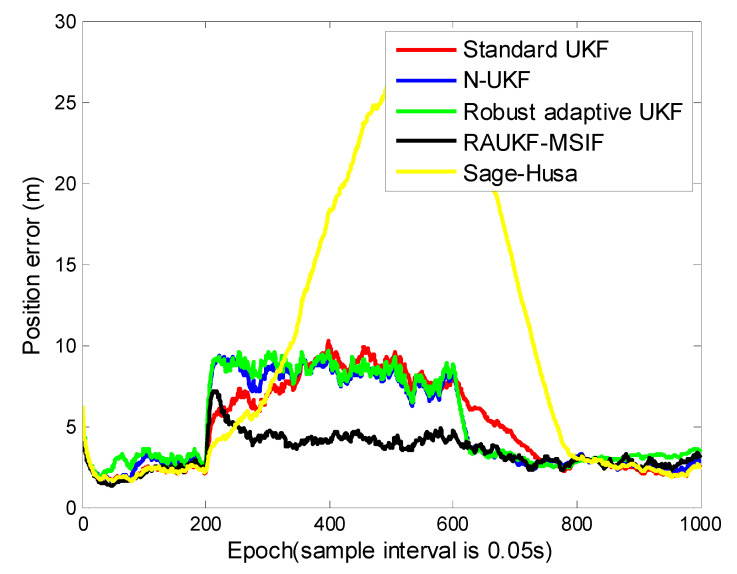
Position tracking errors for Case 2.

**Figure 6:**
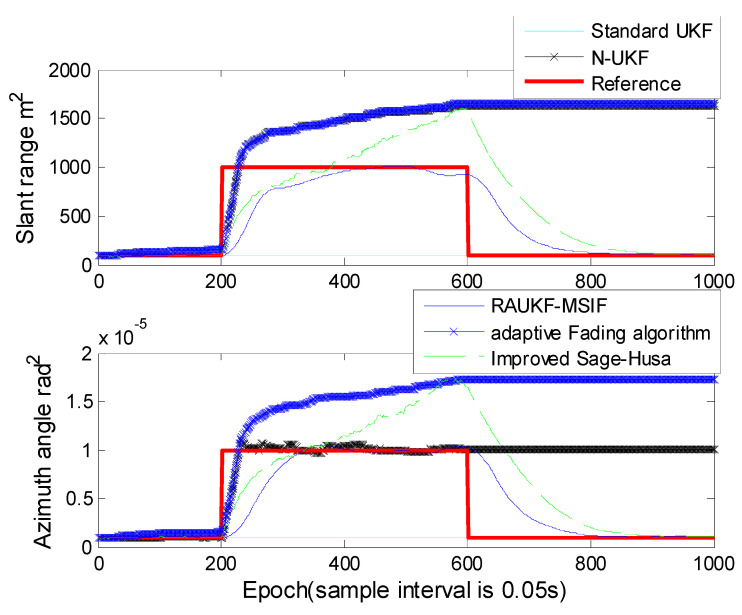
Estimated measurement noise by the algorithms in Case 2.

For Case 2, the improved Sage–Husa adaptive algorithm proposed by Zhang et al. is introduced to verify the performance in R estimation [26]. The position errors estimated by the algorithms mentioned above are shown in (Figure 5), the means and variances of the position tracking errors are listed in Table 2. The measurement noise variances estimated in the algorithms are shown in Figure 6.

It can be seen from Figure 5 and Table 2 that the error of the improved Sage–Husa is much greater than other algorithms. This is because the innovation sequences used by this algorithm are contaminated by the maneuvering motion. The improved Sage–Husa method cannot maintain its accuracy or even diverges when process-error becomes non-negligible, although it can overcome the time-varying noise covariance of the measurement. Similar to N-UKF, the robust adaptive UKF can recognize the mismatch between the theoretical values and the calculated ones of Q and/or R, but it cannot provide the same accuracy as our proposed method because it cannot estimate the R independently.

From Figure 6, we can see that the improved Sage–Husa algorithm could estimate the measurement noise variance. However, this method requires a much longer time to catch up with the reference values; moreover, this method would give a much greater overestimation than ours. The N-UKF and the adaptive fading algorithm can estimate the measurement noise variance only when the variance becomes greater, and they fail when the variance becomes smaller. After the epoch 600, the estimated values cannot be restored to the reference values. The values estimated by the standard UKF are unchanged, because the standard UKF does not have the ability to estimate the measurement noise variance.

**Figure 7:**
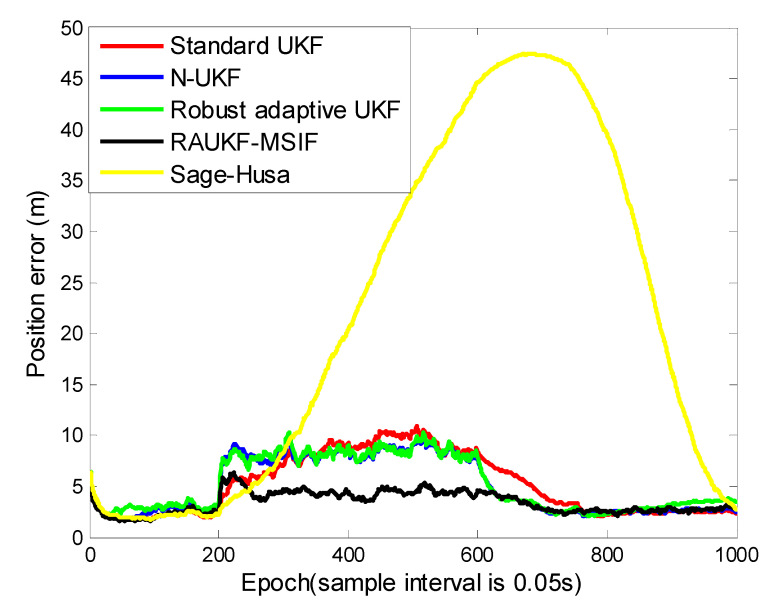
Position tracking errors for Case 3.

For Case 3, from Figure 7 and Table 3, we can see that the standard UKF takes about 200 epochs (from epochs [601, 800]) longer to obtain the accuracy than the N-UKF, the adaptive fading UKF, and our proposed method. The reason is that in order to maintain its accuracy, the standard UKF could only rely on the accurate measurement under the condition in Case 3, this correction procedure has a relative long lag. As to the improved Sage–Husa method, it could be influenced relatively significantly by the process-error more than other algorithms. The simulation result is similar as with Case 2, that the positioning errors estimated by the improved Sage–Husa algorithm are the greatest among the candidates. The N-UKF and the adaptive fading UKF have a similar accuracy because they can both resist the process-error and the time-varying measurement noise to some extent, but they cannot estimate the measurement noise variance as accurate as ours, as mentioned above in Case 2.

### 4.3. Discussion

Base on the decentralized MSIF architecture, we proposed the RAUKF-MSIF to tackle the problems for the nonlinear target tracking systems with time-varying noise covariance. From the results simulated in Case 1, we can see that if there are time-varying noise covariances, the algorithms can detect the process-error and adapt the Q except for the standard UKF.

In practice, the measurement noise covariance is also time-varying. So, in Case 2, we simulate the condition under which each sensors’ measurement noise covariance is set to be 10 times greater during the specified interval than the others. From Figure 6, we can see that the estimation of R by our proposed scheme is more accurate than the others due to RMNCE and the fusion strategy described in Section 2.2 and 3.1. The performance of the improved Sage–Husa is not as good as ours because it could not resist the process-error.

The harshest environment of the simulations is presented in Case 3, in which, together with maneuvering, the matrix Q and R are changed simultaneously in the same interval. As analyzed in Section 4.2 for Case 3, our proposed method can obtain a relatively high accuracy due to both the process-error and the measurement noise variance can be estimated or adapted by our ARUKF-MSIF architecture.

Future work can be divided into two aspects. Firstly, we will focus on improving the numerical stability when adapting the matrix Q. Although the algorithms including ours can adapt the matrix Q, the adapted Q would be a negative definite or the adapted scalar factor would be so much greater than desired that some certain limited values must be set to prevent the algorithms from diverging.

Secondly, in addition to adapting the matrix Q, we would introduce the idea of IMM to estimate the dynamics of the target, while reducing the computational load of the IMM. Due to the existence of redundant measurement, we could improve the accuracy of the probability of the target moving from one model to another. Thus, a more precise model together with an adapted Q would improve the estimation accuracy further.

## 5. Conclusions

This paper addresses the target tracking problem with both time-varying process-error and time-varying measurement noise covariance by using a multi-sensor information filter structure based on an adaptive UKF and the RMNCE. The proposed ARUKF-MSIF employs the RMNCE to estimate the measurement noise variance of each sensor. Subsequently, it uses the theory of hypothesis testing to identify process model uncertainty; next, an innovative algorithm uses both the innovation sequences and the residual sequences to correct the process-error. Then, the algorithm calculates the weight matrices, which are used to fusion the results obtained by each individual sensor. The simulation results demonstrate that the proposed architecture can be robust against the process model uncertainty and error. Moreover, in the harshest circumstances as simulated in Case 3, the proposed method would still maintain the lowest position error among the algorithms. Further, although this paper uses the UKF as the filter, the core idea of the ARUKF-MSIF can be to broaden the EKF, CKF, and other variants of Kalman filter.

## Figures and Tables

**Table 1:** Mean RMSEs of the overall estimation errors for the simulation Case 1. The bold numbers stand for the minimum.

Algorithm	200–600 Epochs		601–1000 Epochs	
	Mean (m)	Variance (m^2^)	Mean (m)	Variance (m^2^)
Standard UKF	4.9253	2.7772	2.7306	0.7438
Robust adaptive UKF	3.1699	0.0620	3.0537	0.0555
N-UKF	3.1010	0.0584	2.9953	0.0552
Our proposed method	**2.7508**	**0.0494**	**2.6785**	**0.0448**

**Table 2:** Position tracking errors of the different algorithms for Case 2. The bold numbers stand for the minimum.

Algorithm	200–600 Epochs		601–1000 Epochs	
	Mean (m)	Variance (m^2^)	Mean (m)	Variance (m^2^)
Standard UKF	7.183457	4.194677	2.963046	0.946813
Robust adaptive UKF	7.516162	4.186171	3.026402	**0.083734**
N-UKF	7.297261	3.885044	2.731655	0.091129
Improved Sage–Husa UKF	15.99712	90.22968	6.232764	34.17588
Our proposed method	**4.160627**	**0.738772**	**2.848997**	0.095412

**Table 3:** Position tracking errors of the different algorithms for Case 3. The bold numbers stand for the minimum.

Algorithm	200–600 Epochs		601–1000 Epochs	
	Mean (m)	Variance (m^2^)	Mean (m)	Variance (m^2^)
Standard UKF	7.477111	5.486144	3.029508	1.137522
Robust adaptive UKF	7.433516	3.756174	2.982606	0.265064
N-UKF	7.350406	3.667636	**2.698935**	0.141301
Improved Sage–Husa UKF	22.03666	240.3023	29.90538	267.7623
Our proposed method	**4.260319**	**0.56904**	2.749053	**0.110879**
